# MHC Class 1 and PDL-1 Status of Primary Tumor and Lymph Node Metastatic Tumor Tissue in Gastric Cancers

**DOI:** 10.1155/2019/4785098

**Published:** 2019-02-06

**Authors:** Ibrahim Halil Erdogdu

**Affiliations:** Adnan Menderes University, Medical School, Department of Pathology, Aydin, Turkey

## Abstract

The prognosis of metastatic gastric cancer is poor. Despite the use of VEGF-, EGFR-, and HER2-targeting agents, prognosis is still poor in advanced gastric cancer. Although cancer immunotherapy responds well in some patients, clinical use is limited due to unanswered patients. For this reason, it is necessary to know the characteristics of primary and metastatic cancer cells for patient selection for immunotherapy and additional criteria are required. MHC-1 downregulation is most frequently observed in the tumor escape mechanism of cancer cells from the immune system. MHC-1 downregulation with increased PDL-1 expression of cancer cells has an important role in immune escape. MHC-1 downregulation and PDL-1 expression have been shown in many types of cancers. However, there is no study on the status of MHC-1 and PDL-1 in primary and metastatic tumor tissue. In this study, MHC-1 and PDL-1 score in primary and metastatic tumor cells was evaluated in 43 gastric cancer patients with lymph node metastasis. According to our results, the primary tumor PDL-1 score was correlated with the number of metastatic lymph nodes (*r* = 0.258; *p* = 0.024) and primary tumor size (*r* = 0.341; *p* = 0.045). A similar correlation was found between the primary tumor PDL-1 score and the metastatic tumor PDL-1 score (*r* = 0.213; *p* = 0.015). In our study, MHC-1 was found to be higher in primary tumors than metastatic tumors, although not statistically significant (*p* = 0.054). The results of our study showed high MHC-1 and low PDL-1 expression in primary tumors and low MHC-1 and high PDL-1 expression in metastatic tumors. These results reveal different biological characteristics of primary and metastatic tumor cells.

## 1. Introduction

Gastric cancer is the third most frequent cause of deaths from cancer in the world [1, 2]. It is usually diagnosed in its advanced stages and has a poor prognosis. Lymph node metastasis frequently appears in most of the cases of gastric cancer. The chance of cure for these cases decreases, and recurrences and distant metastases appear despite treatment. As in all cancer types, knowing the features of metastatic cells is important to determine the treatment for gastric cancer. Metastatic tumor cells can have different phenotypical and biological characteristics from primary tumor cells [3, 4]. The determination of these characteristics is significant to use and develop effective treatment methods. The cancer cells containing numerous genetic and epigenetic abnormalities are eliminated by the immune system. The initiation of the immune response starts with the recognition of the tumor-specific antigens by the major histocompatibility complex (MHC) present on the surface of the antigen-containing cells. The cells that play a central role in the host immune system are the T cells. Following the interaction between MHC and T cell receptors, the immune response is initiated with certain other additional stimuli. It is known that the MHC class 1-positive or heterogeneous tumor cells are eliminated through their recognition by T lymphocytes and even by other immune cells such as the macrophages, whereas the tumor cells representing MHC class 1 downregulation evade the T cell attack. In the case of a so-called “immune escape,” the tumor cells might evade from the host immune system. MHC class 1 downregulation is the most common mechanism of tumor escape from the host immune system. An MHC class 1 downregulation over 90% was reported to be observed in certain types of cancers. This situation might arise as a result of various mechanisms related with the regulation of the immune system. These mechanisms include the downregulation of MHC class 1 expression and the increased expression of immune checkpoint ligands on the cell surface, such as the PDL-1 [5]. In view of the fact that target-specific methods are rapidly developed at present, numerous studies are performed to assess biological markers to evaluate treatment alternatives. Programmed cell death ligand-1 (PDL-1) is one of the target alternatives [6, 7]. PDL-1 is a molecule found in PD-1-activated T cells and limiting and inhibiting immunological activation. Its two ligands which enable this inhibition by binding to PD-1 (PDL-1 and PDL-2) can be found in not only antigen-presenting cells but also tumor cells [8, 9]. Tumors with PD-1 ligand bind to PD-1 in T cells and thus can inhibit the immunological reaction. The monoclonal antibody anti-PD-1 binds to PD-1 and thus prevents binding of ligands. This enables the immunological activation to continue without inhibiting it. The anti-PDL-1 antibody binds to the ligand of PD-1 and to PD-1 and B71 molecules in T cells. This eliminates the inhibition which the ligand activates. It has been reported in the literature that the rate of the response achieved by this monoclonal antibody was higher in the tumors shown to have PDL-1 in their cells immunohistochemically [10, 11]. The drugs exerting their effects by disconnecting the PD-1:PDL-1 pathway forming the immune checkpoint have been approved for the treatment of such diseases difficult to treat such as malignant melanoma, squamous cell pulmonary carcinoma, and renal cell carcinoma due to their successful results [8]. Their effects on many oncological diseases such as Hodgkin lymphoma; bladder, ovarian, gastric, head, neck, colorectal, and pancreas cancer; and cholangiocarcinoma are being investigated [8]. MHC class 1 downregulation is more frequently observed in metastatic cells than is observed in primary tumor cells. It is accepted that the cytotoxic T lymphocytes (CTL) play a key role in tumor eradication and PDL-1 treatment. Taking this into consideration, it becomes of importance that the MHC class 1 expression of tumor cells shall also definitely be considered for a successful PDL-1 treatment.

There have been a few studies on MHC class 1 and PDL-1 in patients with gastric cancer [12–14]. They have focused on PDL-1 in primary tumors. Revealing different biological features of metastatic tumor cells to develop new treatment alternatives will provide additional benefits. Therefore, the present study was directed towards investigating MHC class 1 and PDL-1 in metastatic tumor cells in addition to primary tumors in patients with gastric cancer. To achieve this aim, MHC class 1 and PDL-1 expressions in tumor cells and metastatic lymph nodes of patients with gastric cancer and lymph node metastasis were investigated immunohistochemically.

## 2. Material and Methods

A total of 63 patients diagnosed with adenocarcinoma and operated in Adnan Menderes University Hospital between January 2013 and November 2017 were evaluated. Of 63 patients, 54 had lymph node metastasis. Seven patients were excluded since clinical information about them and their paraffin-embedded blocks could not be accessed, 8 patients were excluded since they had distant metastases, and 5 were excluded since they received treatment before surgery. None of the patients included in the study received chemotherapy and/or radiotherapy before surgery. Ethical approval was obtained from the ethical committee of the university (ADUBAPTPF-2018/1320). Histopathological diagnoses, TNM stages, and clinical features of the patients were recorded. Hematoxylin-eosin-stained preparations of the primary tumors and lymph node metastases were reevaluated, and the paraffin-embedded blocks without necrosis, hemorrhage, or technical deformation were selected to perform immunohistochemical staining.

### 2.1. Immunohistochemistry

All immunostaining was carried out at room temperature by using a DAKO Autostainer Universal Staining System (Autostainer Link 48 DAKO, Glostrup, Denmark). First, sections 4 *μ*m in thickness obtained from selected paraffin-embedded blocks were put on positively charged slides. Second, all the sections were deparaffinized in xylene and dehydrated through a graded series of ethanol solution. Third, antigen retrieval was performed at 96°C (10 mM/L citrate buffer, pH 6) for 40 minutes in a thermostatic bath (PT Link). The sections were incubated with PDL-1 (PDL-1 IHC 22C3 pharmDx, code SK006, DAKO, Glostrup, Denmark) and MHC-1 (HLA-ABC clone W 6/32, code R7000, DAKO, Glostrup, Denmark) for 60 minutes at room temperature. Positive and negative controls were added for each antibody and to each batch of staining. A streptavidin-biotin-enhanced immunoperoxidase technique (K8000 Envision Flex, DAKO, Glostrup, Denmark) in an automated system was used to show immunoreactions. The sections were incubated with DAB and counterstained lightly with hematoxylin to demonstrate binding. Finally, the sections were dehydrated and mounted onto the slides. The tissues known to be positively immunostained were used as positive controls. Normal rabbit serum IgG was used to replace a primary antibody as a negative control.

### 2.2. Evaluation of Immunohistochemical Staining

Immunohistochemical examinations were obtained under a light microscope by a pathologist blinded to their clinical and pathological features.

Immunohistochemical scoring was performed with the semiquantitative method described by Garon et al. [15]. The PDL-1 expression is evaluated by a tumor proportion score (TPS), which is defined as the percentage of viable tumor cells with at least partial membrane staining relative to all viable tumor cells in the examined section. The evaluation of the scores includes partial or complete membrane staining (at least 1+ intensity) that is perceived distinct from cytoplasmic staining. Exclusive cytoplasmic staining should be excluded from the scoring; cytoplasmic staining is seen with membranous staining in most instances. The scoring is interpreted as follows: none—no PDL-1 expression (TPS < 1%), low—PDL-1 expression (TPS 1-49%), and high—PDL-1 expression (TPS ≥ 50%). The immunoreactivity score for MHC class 1 was determined by adding the grade of intensity of the cell membrane (0: no staining, 1: weak, 2: moderate, and 3: strong) and the percentages of positive cells (0: 0, 1: 1–10, 2: 11–30, 3: 31–66, 4: 67–80, and 5: >80%). MHC class 1 expression was classified as high when the immunoreactivity score was ≥5 [16]. Only viable tumor cells are included in the scoring. All other (stained) cells, such as tumor-associated immune cells, normal/nonneoplastic cells, and necrotic cells, should be excluded from the evaluation.

### 2.3. Statistical Analysis

All statistical analyses were made with SPSS (Windows version 13.0, SPSS Inc., Chicago, IL, USA). Continuous variables were compared by Mann-Whitney *U* tests, and categorical data were compared by *χ*^2^ tests. A *p* value of less than 0.05 was considered significant.

## 3. Results

**Figure 1:**
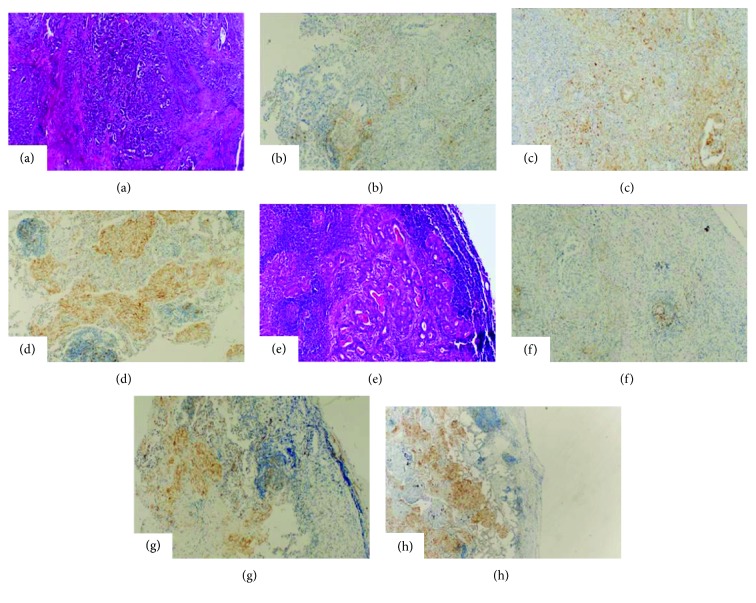
PDL-1 staining in primary tumor and metastatic lymph node in gastric cancer. (a) Gastric cancer primary tumor (HE). (b) Primary tumor (no PDL-1 staining). (c) Primary tumor (low PDL-1 staining). (d) Primary tumor (high PDL-1 staining). (e) Gastric cancer metastatic tumor (HE). (f) Metastatic tumor (no PDL-1 staining). (g) Metastatic tumor (low PDL-1 staining). (h) Metastatic tumor (high PDL-1 staining). All images ×100 magnification.

Clinicopathological features of the patients are presented in Table 1. Most of the patients were male. pT category was found to be pT3 and pT4. No staining with PDL-1 was found in 25 primary tumors (58.1%) and 17 metastatic lymph nodes (39.5%). There was low to high staining in 18 patients with primary tumors (41.9%) and 26 patients with metastatic lymph nodes (60.5%) (Figure 1). The rates of immunohistochemical staining with PDL-1 in primary tumors and metastatic lymph nodes are shown in Table 2.

**Figure 2:**
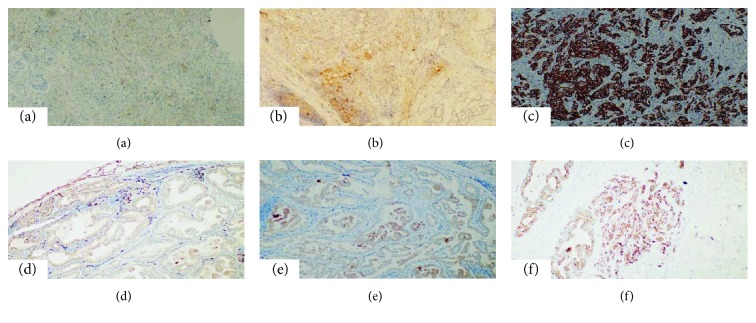
MHC-1 staining in primary tumor and metastatic lymph node in gastric cancer. (a) Primary tumor (no MHC-1 staining). (b) Primary tumor (low MHC-1 staining). (c) Primary tumor (high MHC-1 staining). (d) Metastatic tumor (no MHC-1 staining). (e) Metastatic tumor (low MHC-1 staining). (f) Metastatic tumor (high MHC-1 staining). All images ×100 magnification.

There was a correlation between immunohistochemical PDL-1 staining in the primary tumor and the number of metastatic nodes (*r* = 0.258; *p* = 0.024). Likewise, the size of primary tumor was weakly correlated with PDL-1 staining in the primary tumor size (*r* = 0.341; *p* = 0.045). There was a strong positive correlation between primary tumor PDL-1 staining and metastatic tumor PDL-1 staining (*r* = 0.213; *p* = 0.015). Furthermore, PDL-1 expressions in the primary and metastatic lymph node tissues were not correlated with other clinicopathological parameters, including age, sex, and tumor location. No staining was observed in MHC class 1, 3 primary tumors, and 4 metastatic tumors in our study. High staining was observed for 17 cases (39.5%) in primary tumors and for 13 cases (30.2%) in metastatic tumors (Figure 2). The difference in staining between primary tumors and metastatic tumors was not found as statistically significant (*p* = 0.054). There was no significant relationship between MHC class 1 staining and clinicopathological features. There also did not exist any statistically significant difference between the PDL-1 staining score and the MHC class 1 staining scores (*p* > 0.05).

## 4. Discussion

Gastric cancer is one of the leading causes of deaths from cancers. Although a decrease in its incidence has been reported, it is still lethal [17, 18]. There have been many studies on its development, risk factors, diagnosis, and treatment. In recent years, target-specific treatment alternatives have been utilized for gastric cancer [18]. Cancer cells have abnormal antigen expression due to many genetic and epigenetic anomalies. This helps the immune system to recognize and destroy cancer cells. MHC molecules play an important role for the identification of immune system tumors and presentation of antigens. In this case, T cells function as primary cells. As a result, before cancer clinically appears, cancer cells can be recognized and eliminated. MHC-1 downregulation was demonstrated for many malignant tumors. Though no treatment on this case currently exists, it still is an important target for the immunotherapies since it might be playing an important role in terms of activating T cells against the tumor cells. Decreases in MHC-1 and increases in PDL-1 play an important role in the immune escape of cancer cells. The prognostic significance of MHC class 1 expression was demonstrated by the research conducted on various types of cancer. It was reported that the prognosis was better particularly in tumors with high MHC-1 expression and low PDL-1 and that the MHC-1 was an independent prognostic factor [5]. Among many recent developments in lung cancer treatment are agents directed towards PDL-1. It is thought that PDL-1 expression in tumors can be predictors of biomarkers for treatments directing towards this molecule. So that T lymphocytes can destroy cancer cells successfully, their gap junction connexins should be interlocked. However, if PDL-1, produced by tumor cells, binds to the PD-1 receptor on the surface of T lymphocytes, this interlocking process is prevented. This allows cancer cells to avoid the defense effect of the immune system. The first clinical study on the relation between PDL-1 and cancer by Topalian et al. in 2012 showed that PDL-1 levels could be a predictor of the response to anti-PD-1 treatment [19]. Later, many similar studies were conducted to confirm this finding in other cancer types, especially nonsmall cell lung cancer [20–27]. Research conducted for the treatment of checkpoint blockade frequently failed to consider the state of MHC class 1. There only exist a few studies that investigated the relationship between the state of MHC class 1 and the PDL-1 block therapy. Most of these studies demonstrated that the MHC class 1 downregulation reduced the response to cancer immunotherapy. It was reported particularly in the studies conducted with melanoma and lung cancer that the MHC class 1 analysis was of critical importance for predicting the response to PDL-1 treatment. There also exist studies in the literature reporting that the PD-1/PDL-1 blockade treatment had a high response rate for patients with classical Hodgkin lymphoma having MHC-1 downregulation. These results indicate the complex structure of the antitumor immunity [5, 28–31].

In spite of a decrease in gastric cancer mortality, the five-year overall survival is around 20-30% in patients with advanced stages of cancer, even in those receiving target-specific treatment for HER2 and VEGF receptors [10]. Therefore, attempts to find new treatment alternatives for advanced stages of cancer are underway. Wu et al. performed the first clinical study about the importance of PDL-1 in gastric cancer [32]. They showed the overexpression of PDL-1 in gastric cancer cells. They also suggested that PDL-1 expression was an independent poor prognostic factor and associated with lymph node metastasis, tumor invasion depth, and tumor size. Several other studies carried out later also revealed similar results and showed that PDL-1 expression was present in 40-50% of gastric cancers and had a relation with poor prognosis [33]. However, some recent studies have indicated that it is associated with good prognosis and that its expression differs between Asian and non-Asian cases. In the present study, including patients with local gastric cancer in advanced stages, 41.9% of the patients had PDL-1 expression, which is consistent with the literature. However, high PDL-1 expression, considered as positive, was found in 14% of the primary tumor tissues and 23.3% of the metastatic tumor tissues from the lymph nodes. It was observed in our study that there was a higher presence of PDL-1 expression in the metastatic tumor cells despite the lower presence of MHC-1 expressions. Our results suggest that the presence of a higher MHC class 1 expression in the primary tumor area might indicate that the tumor cells in this area would respond to the immune checkpoint treatments, whereas the tumor cells in the metastatic area might remain unresponsive. The results of our study also revealed that the biological characters of the metastatic tumor cells were different from those of the primary tumor cells. Moreover, it was observed that these different characteristics of the metastatic tumor cells would have an impact on the response to treatment, in immune checkpoint inhibitor treatment. It should be considered that the PDL-1 blockade treatment targeted for primary tumor cells might remain ineffective as a result of increased PDL-1 and decreased MHC-1 expressions of metastatic tumor cells. There has not been an agreement about immunohistochemical scoring of PDL-1 expression yet. While some studies report that the effectiveness of anti-PD-1/PDL-1 treatment is associated with tumor PDL-1 levels, others argue that the treatment effectiveness is independent of PDL-1 levels [34]. PDL-1 expression is evaluated by using tumor proportion scores (TPS). In this scoring system, in which percentages of tumor cells stained with PDL-1 are given, staining at ≥1% is considered positive and staining at ≥50% is considered highly positive. The cut-off values for PDL-1 expression used in clinical studies are 15%, 5%, 10%, and 50%. Despite the lack of a consensus about these values, high PDL-1 expression seems to be better [8, 34–36]. Evaluations of PDL-1 expression are affected by many factors including methodology, cancer treatment, and immune responses of individuals. In the present study, PDL-1 expression was found to be high both in primary and in metastatic tumors with a significant relation between them. In 8 patients with the primary tumor score of none, there was positive staining in metastatic tumor cells. In addition, in all the patients with the primary tumor score of low and high, metastatic tumor cells had PDL-1 expression. No studies demonstrating the PDL-1 and MHC-1 states for primary and metastatic regions in gastric cancer are present. The literature rather contains research demonstrating the relationship between MHC-1 and PDL-1 in lung cancer. Though not statistically significant, the presence of low PDL-1 with high MHC-1 in the primary tumor and the presence of high PDL-1 and low MHC-1 in the metastatic area are notable findings attained in our study. The nonsignificance of the results attained in our study might be stemming from the presence of only a limited number of patients. The presence of the patients having low PDL-1 expression in their primary tumors but high PDL-1 expression in their metastatic areas indicated different biological behaviors of metastatic tumors although the number of these patients was low. It should be kept in mind that weak staining of primary tumors with PDL-1 can be insufficient to predict the effect of anti-PD-1/PDL-1 treatment. Therefore, it should be considered that it may be useful to evaluate the metastatic area for PDL-1 assessment.

In conclusion, immunotherapy has promising results in metastatic gastric cancers that have limited systemic treatment options. It is of utmost importance that these different biological characteristics of metastatic cells shall be monitored in metastatic gastric cancers, while planning the treatment. In terms of patient selection for the effective use of immunotherapeutic agents in these tumors that have considerably limited treatment options, the MHC-1 state shall as well be evaluated along with the PDL-1 state. It is nevertheless important that the existing limitations of the immune checkpoint blockade shall be considered. Despite the progress reported for patients receiving this treatment in terms of OS, ORR, and PFS, many patients still fail to respond to immunotherapy. It was reported that the PDL-1 positive tumors generally had a response rate of 36%. No clear criteria exists for the facilitation of patient selection that would benefit from this treatment. It can thus be recognized that the unresponsiveness of certain tumors to immunotherapy that is currently being increasingly utilized cannot be predicted by the evaluation of the tumor's PDL-1 state alone. The use of antitumor immunity in cancer treatment is a promising and rapidly expanding field. Additional criteria are needed for the standardization and optimization of this treatment. Therefore, further research is required for understanding the mechanisms that direct the response to immunotherapy, for determining the different biological characters of metastatic tumor cells and for increasing the number of patients who will benefit from this treatment effectively.

## Figures and Tables

**Table 1:** Patients characteristics (*n* = 43).

Characteristics	*n* (%)
Age (year)	
Range	34-83
Median	58
Sex	
Male	31 (72)
Female	12 (28)
Tumor size (cm)	
Range	1.8-14
Mean	4 ± 1.7
pT	
T1	0
T2	3
T3	27
T4	13
Dissected lymph node	
Range	8-43
Mean	11 ± 5
Metastatic lymph node	
Range	1-29
Mean	6 ± 1.3

**Table 2:** Association of PDL-1 and MHC-1 expressions between primary tumor and metastatic lymph nodes.

	PDL-1 expression		MHC-1 expression	
Score	Primary tumor	Metastatic lymph node	Primary tumor	Metastatic lymph node
None	25 (58.1%)	17 (39.5%)	3 (7%)	4 (9.3%)
Low	12 (27.9%)	16 (37.2%)	23 (53.5%)	26 (60.5%)
High	6 (14%)	10 (23.3%)	17 (39.5%)	13 (30.2%)
	*p* = 0.015		*p* = 0.054	

## Data Availability

The data used to support the findings of this study are available from the corresponding author upon request.
